# Editorial: Advances and challenges in neonatal surgery: congenital and acquired conditions

**DOI:** 10.3389/fped.2026.1835463

**Published:** 2026-04-10

**Authors:** Simonetta Costa, Irma Capolupo, Laura Valfrè, Augusto Zani

**Affiliations:** 1 Neonatology Unit, Policlinico Casilino, Rome, Italy; 2 Neonatal Intensive Care Unit, Bambino Gesù Children’s Hospital IRCCS, Rome, Italy; 3 Neonatal Surgery Unit, Bambino Gesù Children’s Hospital IRCCS, Rome, Italy; 4 Department of Surgery, Washington University School of Medicine in St. Louis, St. Louis, MO, United States; 5 Division of Pediatric Surgery, Saint Louis Children’s Hospital, St. Louis, MO, United States

**Keywords:** current challenges, future perspective, long-term outcomes, neonatal surgery, novel surgical strategies, surgical decision-making

## Introduction

Congenital anomalies (CA) requiring surgery represent the fourth leading cause of death in children under 5 years of age in 2019, accounting for 9.4% of global deaths (1). Prematurity is a high-risk condition for morbidities, some of which requires surgical intervention, such as necrotizing enterocolitis (NEC) or post-hemorrhagic hydrocephalus (PHH) following intraventricular hemorrhage (IVH). Improving survival of infants with neonatal surgical conditions (NSCs) is challenging, and advances in diagnostic techniques and surgical strategies are needed. To this end, Frontiers developed a Research Topic titled “Advances and Challenges in Neonatal Surgery: Congenital and Acquired Conditions” to focus on different aspects of the management of NSCs.

## Burden of disease and long-term outcomes

CA impact healthcare costs, although the overall financial impact of infants with CA requiring surgery is not fully known. Jeon et al. found that surgical care of CA in infants was associated with significant healthcare utilization, accounting for $4.8 billion (73.4%) of all inpatient charges in 2021 in Texas, despite representing a minority of admissions.

The management of NSCs also requires long-term neurological follow-up and rehabilitative therapy. Lowdermilk et al. reported a high incidence of neurodevelopmental delay documented as early as the first six months of life in infants with esophageal atresia (EA), regardless of gestational age, type of surgery, concurrent anomalies, and frequency of early patient and family support. Similarly, Bai et al. found neurodevelopment problems in infants with congenital diaphragmatic hernia (CDH), highlighting how pulmonary hypoplasia may negatively impact neurodevelopment.

## Necrotizing enterocolitis: clinical relevance, diagnosis and surgical decision-making

NEC is one of the most life-threatening gastrointestinal emergencies in neonates, involving mainly preterm infants, although the presence of CA may trigger its onset, as reported by Zhang et al. and by Wang et al. NEC requires a multimodal evaluation including clinical signs, laboratory markers, and imaging to guide timely and individualized surgical decision-making. Shang et al. identified peritonitis, portal venous gas, and elevated inflammatory markers as independent risk factors for early surgical intervention in neonates with NEC. Particularly, the authors found that interleukine-6 demonstrated the highest predictive accuracy, suggesting its potential value as an objective biomarker for surgical timing.

Limiting resection to necrotic bowel, preserving viable bowel after NEC, is crucial to avoid complications such as anastomotic leakage and short bowel syndrome. Pfennigs et al. suggested that indocyanine green fluorescent imaging could be a reliable method for visualizing intestinal perfusion in real time, improving the assessment of intestinal viability during laparotomy, overcoming the limitations of standard visual inspection.

## Surgical strategies and innovation in perioperative management

Temporary enterostomy serves as a crucial surgical procedure for treating several NSCs. Despite advancements in surgical techniques and perioperative management, complications related to the enterostomy still remain high. Zeng et al. identified smaller weight at surgery, small intestinal stoma, and longer duration of postoperative high-level C-reactive protein as independent risk factors for enterostomy-related complications and constructed a risk prediction model in infants with stomas.

Focusing on NEC, Tomaselli et al. provided a comprehensive review on the use of mesenchymal stromal cells and their derivatives, such as exosomes or secretomes, for the treatment of this neonatal intestinal emergency, emphasizing the need to generate more robust and comparable results to facilitate their translation to the bedside.

Liu et al. observed that robotic-assisted surgery can significantly improve efficiency in the more complex suturing steps during hepaticojejunostomy, also offering a smoother learning curve compared to conventional laparoscopic surgery. Although this observation was based on a retrospective study, it highlighted the potential advantage of robotic-assisted surgery for complex paediatric procedures.

Neonatal thoracoscopic repair of EA requires one-lung ventilation (OLV), which requires finding strategies to limit volotrauma and barotrauma. Kaimin et al. compared pressure-controlled ventilation with volume guarantee (PCV-VG) with conventional volume-controlled ventilation (VCV) for improving respiratory outcomes during OLV, observing that PCV-VG offered better ventilation parameters and faster recovery in thoracoscopic repair of neonatal EA, while not affecting postoperative complication rates.

Brandau et al. reported a case that highlighted the potential complications associated with thoracoscopic clip closure of tracheoesophageal fistula, showing an alternative closure through the application of endoscopic transfixing sutures.

Krahulik et al. presented their single-center experience with the surgical management of PHH in premature infants, hypothesizing a possible advantage of ventricular access devices over other temporary surgical measures, such as ventriculosubgaleal shunts and external ventricular drainage, in terms of neurological outcomes.

## Prenatal diagnosis and fetal-neonatal planning

Zhang et al. shared their experience with prenatal ultrasound (US) diagnosis of fetal cervical masses, highlighting how prenatal US can accurately assess the size and location of the cervical mass and dynamically monitor fetal airway pressure and amniotic fluid flow. Prenatal US, combined with fetal MRI, seemed to be useful for choosing the mode of delivery, such as the ex-utero intrapartum treatment (EXIT) and planning postpartum management.

The EXIT procedure is a surgical technique used during caesarean section to perform life-saving fetal interventions while maintaining placental circulation. A narrative review by Gaffuri et al. emphasized that the success of EXIT depends on accurate prenatal diagnosis via fetal US and MRI, and that neonatal mortality and complications vary depending on the indications and postnatal management.

Zhuang et al. aimed to identify risk factors for significant liver fibrosis in prenatally diagnosed choledochal cysts (CDCs), identifying type IV CDCs and GGT > 327 U/L as risk factors suggesting the need for close clinical monitoring and timely surgical intervention.

Wang et al. reported their experience with two-dimensional and three-dimensional prenatal US diagnosis of congenital ear anomalies. They found that prenatal US can be useful for identifying fetal ear deformities and associated craniofacial anomalies, representing a valuable tool for providing essential information for clinical management and prenatal counseling.

## Disease-specific surgical considerations

The appropriate length of dilated segment resection (DSRL) in Hirschsprung's disease (HSCR) remains a matter of debate, and its correlation with postoperative clinical outcomes has not yet been clarified. Jia et al. explored the relationship between DSRL and short-term clinical outcomes in HSCR. The authors found that not only DSRL, but also the length of aganglionosis, were relevant to outcomes. For short-segment HSCR, DSRL, total length of bowel resection and length of aganglionosis did not show a significant impact on short-term clinical outcomes. In contrast, these parameters in long-segment HSCR were significantly associated with fecal incontinence and perianal erosion, although overall patient quality of life remained satisfactory. Furthermore, the results of this study suggested that optimal clinical outcomes were associated with a DSRL of 7.25 cm for short-segment HSCR and 13.00 cm for long-segment HSCR.

Youssef et al. observed that delayed extubation in infants undergoing extramucosal pyloromyotomy was associated with metabolic alkalosis, hypothesizing that metabolic disturbances, particularly alkalosis, may influence respiratory drive.

## Multidisciplinary management and rare neonatal surgical conditions

Zhou et al. and Cai et al. emphasized that multidisciplinary management is essential to address life-threatening neonatal surgical conditions, such as extrarenal malignant rhabdoid tumor and lymphatic malformations with an epiglottic cyst.

The neonatal period often presents surgical challenges for uncommon diseases. For this reason, the Research Topic included space for the presentation of relevant case reports, such as those concerning hepatopulmonary fusion, a rare congenital malformation often associated with right-sided CDH. Given the rarity of this condition, clear guidelines for the optimal management are lacking. The case reports by Tedesco et al. and Kanda et al. represent a valuable contribution to the limited literature on this NSC. These case reports highlighted the importance of preoperative imaging to assess the vascularization in order to decide whether to separate the liver and lung. Even when the vascularization allows for separation, the postoperative period may not be free of complications, including airway complications.

Congenital imperforate hymen is a rare anomaly that often remains asymptomatic until adolescence, when patients typically present with primary amenorrhea, cyclic abdominal pain, and hematocolpos. Bian et al. described a case of a fetus with prenatal US-detected fetal pelvic cystic mass, which was confirmed by MRI, who underwent prenatal US-guided aspiration and drainage, followed by successful postnatal surgical treatment for hymenal atresia.

Chen et al. focused on hematochezia and hematemesis as possible early clinical signs of volvulus due to intestinal malrotation in the neonatal period. The clinical case described by Tvildiani et al. demonstrated the feasibility of temporary dual-site epicardial pacing, followed by delayed permanent implantation, in an extremely premature infant with complete atrioventricular block.

Progressive exophthalmos following minor trauma is a rare condition and it is important to consider subgaleal hematoma in the differential diagnosis. The case reported by Lee et al. suggested that a high clinical suspicion after head trauma and an early diagnosis of subgaleal hematoma can prevent permanent vision loss due to optic nerve damage or pressure-induced corneal damage.

## Additional contributions in paediatric surgery

Finally, the Research Topic gave space to some research in the field of paediatric surgery. Gu et al. retrospectively examined 25 children diagnosed with type III biliary atresia who underwent laparoscopic Kasai surgery, with regard to whether or not they had received a partial quadrate lobectomy**.** The authors observed that partial quadrate lobectomy during laparoscopic Kasai surgery may reduce operative time, decrease the incidence of cholangitis, and improve jaundice resolution rates without increasing intraoperative blood loss or negatively impacting one-year native liver survival.

Qin et al. retrospectively analysed the outcomes of 128 children with acute appendicitis who underwent a novel transumbilical double-port single-port laparoscopic appendectomy (TSSDPLA) or a traditional triple-port laparoscopic appendectomy (TPLA). The TSSDPLA technique retained the advantages of TPLA, while reducing the need for specialized instruments and consequently lowering surgical costs. Furthermore, it improved postoperative cosmetic outcomes by eliminating visible scarring.

Chen et al. conducted a systematic review to explore the optimal treatment approach for perianal abscess (PA) and anal fistula (FAI) in children. Available studies, including 1,770 patients, indicated minimal differences in the healing and recurrence rates of PA and FAI between the conservative and surgical treatment groups. The authors, however, emphasized that these findings are based on retrospective studies with small sample sizes and short follow-up periods, and called for further high-quality studies to establish optimal treatment protocols.

In a case series, Ben et al. reported the results of automated endothelial keratoplasty with Descemet membrane removal (DSAEK) for paediatric Descemet membrane detachment (DMD). The authors found that paediatric DMD may have a lower success rate with descemetopexy due to anatomical peculiarities and that reconstruction of the visual pathways to promote early visual development justifies a more aggressive treatment such as DSAEK, which appeared to offer promising results.

## Conclusion

This Research Topic received 42 submissions, 69% of which were accepted for publication. The attention this Research Topic has generated is a sign of the considerable scientific interest that drives research in neonatal and paediatric surgery.
